# Derived Time in Range and Other Metrics of Poor Glycemic Control Associated With Adverse Hospital Outcomes in Patients With Diabetes Mellitus Admitted to Non-ICU Wards at a Tertiary-Level Hospital in Colombia: A Cross-Sectional Study

**DOI:** 10.1155/2024/3451158

**Published:** 2024-08-27

**Authors:** Edwin Mora Garzón, Alexandra González Montoya, Gilma Hernández Herrera

**Affiliations:** ^1^ Division of Endocrinology and Metabolism Department of Internal Medicine and Epidemiology S.E.S. University Hospital of Caldas University of Caldas, Manizales, Colombia; ^2^ Department of Internal Medicine University of Caldas, Manizales, Colombia; ^3^ Department of Epidemiology University of Rosario, Bogotá, Colombia

**Keywords:** diabetes mellitus, glycemic control, inpatient, outcome assessment

## Abstract

**Aim:** This study is aimed at assessing the prevalence of poor glycemic control using different metrics and its association with in-hospital adverse outcomes.

**Methods:** This cross-sectional study was conducted in diabetic patients admitted to a third-level hospital in Colombia between January and July 2022. Poor glycemic control was determined using capillary glucose metrics, including mean glucose values outside the target range, derived time in range (dTIR) (100–180 mg/dL) < 70%, coefficient of variation (CV > 36%), and hypoglycemia (<70 mg/dL). Multiple regression models were adjusted for hospital outcomes based on glycemic control, as well as other sociodemographic and clinical covariates.

**Results:** A total of 330 Hispanic patients were included. A total of 27.6% had mean glucose measurements outside the target range, 33% had a high CV, 64.8% had low dTIR, and 28.8% experienced hypoglycemia. The in-hospital mortality rate was 8.8%. An admission HbA1c level greater than 7% was linked to an increased mortality risk (*p* = 0.016), as well as a higher average of glucometer readings (186 mg/dL vs. 143 mg/dL; *p* < 0.001). A lower average of dTIR (41.0% vs. 60.0%; *p* < 0.001) was also associated with a higher mortality risk. Glycemic variability was correlated with an increased risk of mortality, hypoglycemia, delirium, and length of hospital stay (LOS).

**Conclusion:** A significant number of hospitalized diabetic patients exhibit poor glycemic control, which has been found to be associated with adverse outcomes, including increased mortality. Metrics like dTIR and glycemic variability should be considered as targets for glycemic control, highlighting the need for enhanced management strategies.

## 1. Introduction

Diabetes mellitus (DM) is a disease with a high global prevalence that results in substantial morbidity and mortality, thereby posing a significant social and economic impact on the healthcare system [1]. It is estimated that approximately 9% of the world's population has diabetes, and this prevalence increases to 19.9% among adults aged between 75 and 79 years [2]. The high prevalence of DM often makes it a frequent condition encountered in emergency rooms and inpatient settings. Studies indicate that about 22% to 46% of hospitalized patients have diabetes, which significantly contributes to the high costs incurred by healthcare systems. In fact, hospital care could potentially account for 40% of total medical expenses [3]. During hospitalization, poor glycemic control—whether due to hyperglycemia, hypoglycemia, or glycemic variability—is associated with adverse clinical outcomes, including infections, extended length of hospital stay (LOS), admission to the intensive care unit (ICU), early readmission (within 30 days), and death [1, 4–7].

Various studies have established the operational definition of poor glycemic control as a single glucose record above a set value, as well as a high mean of capillary glucose levels, regardless of whether the patient is in general wards, preoperatively or postoperatively. Recently, other metrics of glycemic control, such as glycemic variability and time in range (TIR), have been introduced. These are measured by continuous glucose monitoring (CGM) or derived (dTIR) from capillary glucose measurements [8–10].

This study is aimed at establishing the prevalence of poor glycemic control using different operational definitions and their association with adverse in-hospital outcomes.

## 2. Methods

This cross-sectional study was conducted at a tertiary hospital in Manizales, Colombia. We evaluated records of patients 18 or older with DM, hospitalized for medical or surgical reasons between January and July 2022. Included were DM1 or DM2 diagnosed patients, regardless of HbA1c value, presence or absence of macro or microvascular complications, or hospitalization reason. Those with incomplete medical records, no HbA1c report, hospitalization under 2 days, pregnancy, or using insulin pumps were excluded. Glycemic control was determined by capillary glucose reports. Good control was defined as a mean glucose reading of 100–180 mg/dL, dTIR of 100–180 mg/dL over 70% of the time, CV under 36%, and no hypoglycemia instances (glucose meter value less than 70 mg/dL). Given the constraints of implementing CGM in numerous hospitals within developing countries and drawing from the research conducted by Lachin et al., Avari et al., and Bergenstal et al. [8–10], which established and assessed TIR using capillary blood glucose monitoring (CBGM), we aimed to explore the association between TIR derived from recorded glucometer readings and in-hospital outcomes. Additionally, considering existing approaches to glycemic variability assessment using self-measured blood glucose, as demonstrated in Zinman et al.'s study [11] among other studies [5, 12–14], we decided to conduct an analysis based on CBGM records (determining the %CV and the standard deviation) and their association with various clinical outcomes. The project received approval from the Clinical Department and Bioethics Committee of the University of Caldas, as well as the Clinical Research Department and Bioethics Committee of the SES University Hospital de Caldas. As this study was classified as nonrisk in accordance with national regulations, patient informed consent was not required. The confidentiality of the medical records was strictly maintained as per the documents provided by the researchers.

### 2.1. Statistical Analysis

This study utilized nonprobabilistic, convenience sampling, estimating a sample size of 330 patients for a 95% confidence level and 5% absolute precision. We calculated central tendency measures (mean/median) and dispersion (standard deviation/interquartile range) for continuous sociodemographic and clinical variables. For qualitative variables, we made frequency tables showing absolute numbers and percentages. Statistical methods were used to estimate the prevalence of poor glycemic control at the institution. To identify factors associated with poor metabolic control, we used chi-square or Fisher's exact tests based on assumption compliance. Additionally, we employed logistic regression to estimate adjusted associations from previous test results. To determine differences in length of stay between well-controlled patients and those not, we conducted mean or median comparison tests based on assumption compliance. Also, we used chi-square tests to compare proportions of infection, death, or readmission among patients, estimating 95% confidence intervals for these differences. Additionally, odds ratios (OR) were calculated to estimate the magnitude of the association between glycemic control and infection, death, LOS, and readmission of patients with diabetes. Finally, multiple regression models were adjusted for readmission, infection, mortality, and LOS outcomes, based on glycemic control and other sociodemographic and clinical covariates. The statistical package R 4.2.2 and its graphical interface R Studio 2022.02.1 were used for analysis.

## 3. Results

### 3.1. Patient Characteristics

A total of 330 Hispanic patients were included. The average age was 69 years (SD 14.7) with a range of ages between 18 and 97 years and a similar distribution between men (48%) and women (52%). The average BMI was in the overweight range (27.2 kg/m^2^ SD 5.41), and about 1 in 5 patients (22%) was in the obese range. Most patients were diagnosed with Type 2 DM (95.2%). Regarding vascular complications derived from diabetes, 56.3% (186) of the patients presented at least 1 diabetic complication, 43.6% (144) at least 1 microvascular complication, and 36% (119) at least 1 macrovascular complication, with the most representative being diabetic nephropathy in 39%, followed by coronary artery disease in 23% (Table 1).

### 3.2. Hospital-Level Description

The average HbA1c level at admission was 8.17% (SD 2.63). The main cause of admission was infection (35%). Approximately one in five patients (19%) required admission to the ICU, and 35% had a history of hospitalization in the past year. The median LOS was 9 days (range 2–80 days). The most used management scheme during hospitalization was the sliding-scale insulin regimen (47.1%), with rapid-acting analogs being the most common (69%), (Table 1).

### 3.3. Hospital Glycemic Control

A total of 58.2% of the patients were admitted with an HbA1c level > 7%. After intrahospital management, nearly one in four patients had a mean of capillary glucose readings outside the target range (27.4%). A total of 64% of patients had low TIR (dTIR < 70%), with an average percentage of dTIR of 58.1%. Additionally, 33% of patients had high glycemic variability (%CV > 36%). (Table 2).

### 3.4. Hypoglycemia

Hypoglycemic events occurred in 28.8% of the patients (Table S1). Level 2 hypoglycemia was reported in 8.8% of patients (Table S2). Upon evaluating hypoglycemia using dTIR, a high percentage of time (15%) was found to be below 100 mg/dL. When comparing %CV between the group of patients with and without hypoglycemia, a statistically significant difference was found (38% vs 25.8%, *p* < 0.001). Similarly, a statistically significant difference was found when comparing the S. D between these groups (59 mg/dL vs. 38 mg/dL, *p* < 0.001) and a lower dTIR in the hypoglycemic group (47% vs 66%, *p* < 0.001).

### 3.5. Hospital Adverse Outcomes

Hospital mortality occurred in approximately 1 in 11 hospitalized diabetic patients (8.8%); admission to the ICU in 11.2%, and early readmission in 22.1%. Among the other outcomes evaluated, delirium, acute kidney injury, and hospital-acquired infections were the most prevalent (Table 3).

### 3.6. Factors Associated With Poor Glycemic Control

In the analysis of variables associated with metrics of poor glycemic control, it was found that the HbA1c level at admission was associated with all four analyzed metrics. Patients admitted for acute complications of diabetes and CKD were found to have a higher likelihood of having poor glycemic control. Finally, higher BMI was associated with less glucose variability (Table S3).

### 3.7. Poor Glycemic Control and Adverse Outcomes

**Figure 1:**
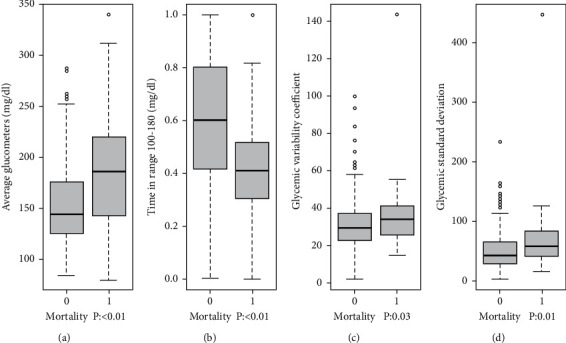
Graphs with quantitative metrics of glycemic control and mortality. rOR, raw odds ratio; aOR, adjusted OR for age, BMI, type of diabetes, reason for admission, length of hospitalization, heart failure, coronary disease, chronic kidney disease, CHF, and COPD. (a) Mean glucometries rOR 1.0152 (CI95% 1.007–1.023) *p* < 0.01, aOR 1.019 (CI95% 1.009–1.029) *p* < 0.01. (b) Time in ranges rOR 0.023 (CI95% 0.003–0.133) *p* < 0.01 aOR 0.013 (CI95% 0.001–0.094) *p* < 0.01. (c) Glycemic variability coefficient rOR 1.022 (CI95% 1.001–1.044) *p* 0.03 aOR 1.018 (CI95% 0.994–1.043) *p* 0.13. (d) Glycemic standard deviation rOR 1.012 (CI95% 1.002–1.021) *p* 0.01. aOR 1.013 (CI95% 1.001–1.026) *p* 0.03. HbA1c rOR 1.033 (CI95% 0.903–1.183) *p* 0.63.

When evaluating the relationship between different metrics of glycemic control and adverse outcomes, a significant association was found between mortality and the mean of capillary glucose readings outside the target range, HbA1c, and low dTIR, in both bivariate and multivariate analyses (Table 4). When including the mean of capillary glucose readings as a quantitative variable in the logistic model, it was observed that an increase in the mean significantly increased the probability of mortality (aOR 1.019, 95% CI 1.009-1.029, *p* < 0.001), implying that for every 1 mg/dL increase in the mean of capillary glucose measurements, the probability of mortality increased by approximately 1.9% (Figure 1). The median for this indicator in patients who died versus those who did not was 186 mg/dL vs. 143 mg/dL (*p* = 0.002). When dTIR was included as a quantitative variable in the logistic model, a negative association was observed with mortality (aOR 0.013, 95% CI 0.001–0.094, *p* < 0.01), indicating that greater dTIR leads to lower mortality. The median dTIR in the mortality group was 41% versus 60% in the nonmortality group (*p* < 0.01). In the unadjusted quantitative analysis, the dTIR and %CV showed opposite associations with mortality. dTIR showed an inverse relationship with mortality (for each point where dTIR increased, the probability of mortality decreased by approximately 2%), whereas %CV showed a direct relationship with the probability of mortality (for each point where CV increased, the risk increased by approximately 2%) (Figure 1). For early readmission, an association was found with the mean of capillary glucose readings when they were slightly outside the target range (*p* = 0.03); however, this association was lost when the average was above 200 mg/dL. For hospital-acquired infection, only a high %CV and hypoglycemia were associated in the bivariate analysis (*p* = 0.01) (Table 4). The median %CV in the infected group was 33.5% compared to 28.3% in the noninfected group (*p* = 0.01).

### 3.8. Variables Associated With Hospital LOS

After evaluating LOS with different metrics, including the average of capillary glucose measurements, %CV, dTIR, and HbA1c, the only statistically significant variable was %CV with a beta of 0.076 (95% CI 0.0021–0.14 and *p* = 0.043), implying that for every unit increase in %CV, the average hospital length of stay increases by 0.07 days.

Hypoglycemia was another variable that was found to be statistically significantly associated with LOS. For the group that had Level 1 hypoglycemia (<70 mg/dl) compared to those who did not, the median LOS was 12 vs. 8 days (*p* < 0.01). The median LOS was 13 days in the Level 2 hypoglycemia group (<54 mg/dL), (Table 5).

## 4. Discussion

This study has the strength of evaluating four different glycemic control metrics and their association with adverse in-hospital outcomes. To our knowledge, it is the first investigation to specifically explore this association through the use of dTIR in the setting of non-ICU hospitalizations. Additionally, it is a pioneer in our country in reporting the prevalence of inadequate glycemic control and its relationship with negative clinical outcomes. When poor glycemic control was established by the mean of capillary glucose readings, the prevalence was 27.6%, established by %CV 33%, and 64.8% by low dTIR, with hypoglycemia occurring in 28.8% of the cases. Variables associated with poor hospital glycemic control were admission HbA1c, CKD, BMI, and the cause of hospital admission.

When compared to reference publications, in one study by Cook et al., [15], in North American hospitals, an average of 166 mg/dl (SD +/−8) was reported in non-ICU wards. An average of 26.3% of patients had >180 mg/dL. In a study by Swanson et al., the average was 166 mg/dL (SD +/−11), with 32% of patients having averages > 180 mg/dL [16]. In our study, the average was 156 mg/dL with a high SD of +/−42.5. High average glucose levels have been widely associated with adverse outcomes in the hospital setting, including death [1, 5, 17, 18]. Hence, current guidelines [1] recommend maintaining an average glucose level between 100 and 180 mg/dL. Consistent with this, our study found that the average glucose level among patients who died versus those who did not was 186 mg/dL (95% CI 143−220) versus 143 mg/dl (95% CI 125–176), *p* < 0.001. This underscores the importance of adhering to these recommendations.

Glycemic variability at the hospital level has also been associated with adverse clinical outcomes [5, 11–14, 19]. Nair's study [5] reported an association with increased mortality risk (OR = 1.42; 95% CI: 1.14 to 1.71; *p* = 0.003) and prolonged hospital stays (an additional 0.32 days for every 10 mg/dL increase). Similarly, Jordán-Domingo's research [12] indicated an increased risk of mortality (HR = 1.52; 95% CI: 1.12-2.06; *p* = 0.006). In Kim's ICU study [13], a CV higher than 36% was identified as an independent risk factor for mortality and extended ICU stays. Méndez reported that an increase of 10 mg/dL in the SD and a 10% increase in the CV resulted in increases of 4.4% and 9.7% in the LOS, respectively. Additionally, the report highlighted that each 10 mg/dL increase in SD was associated with an 8% increase in mortality [19]. In our study, glycemic variability, as determined by the SD, was higher in patients who died (SD 57.5, 95% CI 40.4−83.4) compared to those who survived (SD 41.6, 95% CI 28.6–65.0), *p* = 0.007.

dTIR has been investigated in the ICU setting for both diabetic and nondiabetic populations, across various established ranges and percentages. Hiromu Naraba's study [20] established a TIR of 70–180 mg/dL based on measured blood glucose values (3 times per day). It was found that in patients with HbA1c levels below 6.5%, a TIR below 80% was associated with an increased risk of 28-day mortality, with an adjusted OR (aOR) of 1.88 (95% CI: 1.36–2.61). However, this association was not observed in patients with HbA1c levels above 6.5%. In the study conducted by Okazaki et al. [21] with an average of 14 blood glucose measurements per patient, establishing a TIR of 70–140, it was reported that a TIR above 80% was linked to reduce in-hospital mortality (adjusted OR 0.16; 95% CI 0.06–0.43; *p* < 0.001). There is a lack of data on dTIR in non-ICU wards; in our study, we found that a dTIR below 70% was associated with higher mortality (aOR 7.57; 95% CI 2.09−27.3; p 0.002). Furthermore, among patients who survived, the average dTIR was higher compared to those who died (60%, 95% CI 41.7-80.0 vs. 41%, 95% CI 30.0-51.0, *p* < .001).

In our study, mortality was reported in 8.8% of the patients, and we found that a mean capillary glucose level outside the target range (established as 100 to 180 mg/dL), a low dTIR (less than 70% between 100 and 180 mg/dL), and a high HbA1c level (more than 7.0%) were statistically significantly associated with mortality. Additionally, after quantitative analysis, both %CV and SD were also associated with mortality. The %CV and TIR showed opposite associations with mortality in the quantitative analysis (dTIR had an inverse association, reducing the probability of mortality by 2% for every point increase, whereas %CV had a direct association, increasing the risk by 2% for every point increase). Compared with international publications, Umpierrez et al. [17] reported a mortality rate of 3% with a mean hospital stay of 5.5 (SD +/−0.2) days, while Russo et al. [22] reported a mortality rate of 7.6% with a median hospital stay of 5.1 days and hypoglycemia (less than 70 mg/dL) in 10.2% and severe hypoglycemia (less than 40 mg/dL) in 1.7% of patients. In a study by Ferreira on diabetic patients with COPD or pneumonia, the reported mortality rate was 7.4%, with a median hospital stay of 10 days and a low number of capillary glucose measurements per day (2.1). The study reported a 13.4% incidence of hypoglycemia and a mean %CV of 30.7 ± 6.8 with a statistically significant association with hospital stay [23]. In another publication [24], the mortality rate was 7.1%, with a mean hospital stay of 4.9 days; however, there are publications [25], reporting a mortality rate of 11.2% with a median hospital stay of 6 days, where the most frequent cause of death was cerebrovascular disease (41.6%), followed by infections (23.1%).

Our proportion of patients requiring readmission within 30 days was 22.1%, falling within the highest range reported in the literature, which has been described as ranging from 14.4% to 22.7% [26]. Of our patients, 35% had at least one previous hospitalization, which is slightly higher than what has been reported in the literature, where approximately 30% require two or more hospitalizations within a given year [18].

%CV and hypoglycemia were found to be associated with infection, as previously reported in the literature [27]. It has also been reported that in the early stages of sepsis, the onset of hypoglycemia may be associated with disease severity [28]. In our study, hypoglycemia was reported in 28.8% of patients, although it has been reported to range from 1% to 33%. These findings are concerning, given the potential to cause harm by increasing the risk of serious adverse events, such as seizures, permanent brain damage, cardiac arrest, and death [3–6]. However, in our study, we did not find an association with mortality, probably due to the lack of power and a single observation. Additionally, the prevalence of hypoglycemia is likely underestimated due to the limited number of capillary glucose tests performed. This issue, along with other variables, leads to varied reports across studies regarding the prevalence of inpatient hypoglycemia. We found in the bivariate analysis that hypoglycemia was associated with delirium, ICU admission, and a longer hospital stay (increasing hospital stay by 4 to 5 days).

Regarding outpatient management, it is noteworthy that in the face of the proportion of patients with vascular complications, the use of iSGLT2 or aGLP1 is lower, as is the case with statins, which implies noncompliance with clinical practice guidelines. In intrahospital management, we identified a lack of a structured program for hospitalized diabetic patients, widespread use of sliding scale insulin in 47% of cases, contrary to standard guidelines, and inconsistent glucose monitoring. Current guidelines recommend monitoring glucose before meals and every 4 to 6 h for patients who are not eating. However, this leads to significant variability in the records reported and published across different studies, with daily glucose tests generally ranging from about three, as observed in the RABBIT 2 Surgery study [29], to between 2 and 4 measurements per day [5, 12, 19–21, 23]. Such inconsistency could hinder the effective detection of glycemic variability and hypoglycemia, potentially leading to underestimations [30]. In light of our study findings and literature, a recommendation of at least 4 glucometries per day (before meals or in the main meal segment and at bedtime) and ideally 7 glucometries per day (including 2 h postprandial), along with additional assessments in cases of suspected or documented hypoglycemia, could improve record accuracy and clinical decision-making.

### 4.1. Limitations

Limitations were found in data collection due to some variables not being consistently recorded in the medical records, which limited their inclusion in the analysis (duration of diabetes, Level 3 hypoglycemia recording, microalbuminuria). In our study, CGM was not used to measure the time in therapeutic ranges or glycemic variability. However, the correlation between TER by MCG and capillary glucose measurement is good [8], but not with glycemic variability [5]. Traditionally, dTIR has been derived from 7-point daily glucose profiles. In our study, the median number of daily capillary monitoring was 2.9 (2.0–4.0). In contrast, the median number of measurements per patient was 27 (16–49), with a total of 8519 blood glucose tests conducted. Aware that the frequency of capillary glucose is an important factor that can influence the metrics analyzed in our study, future research should consider the use of CGM to contrast the results with those obtained through capillary glucose tests. This approach would yield more robust and precise data, enhancing our understanding of glycemic control and its clinical implications in hospitalized diabetic patients. Mortality and readmission outcomes may be underestimated due to the punctual evaluation of the event considering the type of study. In the case of readmission, underestimation could also occur because some patients are readmitted to other institutions in the city.

## 5. Conclusions

Our findings indicate that a substantial proportion of patients experience suboptimal glycemic control, as evidenced by an average outside of goals, low TIR, high glycemic variability, and episodes of hypoglycemia. The significant association between poor glycemic control and an increase in hospital mortality underscores the importance of effective glucose management in this patient group.

These results highlight the imperative need for intervention strategies to improve glycemic control in hospitalized patients with diabetes, which could significantly enhance health outcomes. The metrics used in the study, such as the %CV and the dTIR, prove to be valuable tools in the assessment and goal-setting for glycemic control. Our study provides a foundation for future research and clinical practices aimed at optimizing diabetes management in the hospital setting.

## Figures and Tables

**Table 1:** Demographic and clinical characteristics of the participants in the study at the time of their admission and hospitalization (*n* = 330).

**Baseline characteristics**	**n** = 330	**In hospital characteristics**	**(** **n** = 330**)**
Age, mean (SD) minimum–maximum (years)	69.9 (14.7) 18–97	HbA1c at admission % (SD)	8.17 (2.63)
Women, *n* (%)	174 (52.7)	Median LOS, days (Q1–3) min–max	9 (6–15) 2–80
BMI, mean (SD)	27.2 (5.41)	ICU admission, *n* (%)	63 (19)
BMI-defined obesity, *n* (%)	75 (22)		
Type of diabetes *n*/total *n* (%)		Mean admission creatinine (SD)	1.57 (1.89)
Type 1	16/330 (4.8)	<60 ml/min/1.73 m2 *n* (%)	135 (40.9)
Type 2	314/330 (95.2)	<30 ml/min/1.73 m2 *n* (%)	55 (16.7)
DM complications, at least 1, *n* (%)	186 (56.3)	Admission causes^a^*n* (%)	
Diabetic nephropathy	129 (39.1)	Infectious	117 (35.5)
Diabetic neuropathy	44 (13.4)	MI or CVD	33 (10.4)
Diabetic retinopathy	19 (5.8)	Acute complications of DM	32 (9.7)
Diabetic gastroparesis	5 (1.5)	Surgery	29 (8.8)
Coronary artery disease	78 (23.6)	AHF	21 (6.4)
Cerebrovascular disease	42 (12.7)	COPD	19 (5.8)
Peripheral arterial disease	32 (9.7)	Others	79 (23.9)
Medical history, *n* (%)		Treatment regimen, *n* (%)	
Hypertension	255 (77.3)	Corrective only	155 (47.1)
Dyslipidemia	231 (70)	Basal bolus + correction	113 (34.3)
CKD	129 (39.1)	Basal insulin + correction	23 (7)
Hypothyroidism	100 (30.3)	Oral antidiabetic + correction	13 (4)
COPD	78 (23.6)	Basal bolus without correction	10 (3)
CHF	89 (27)	Oral antidiabetic only	5 (1.5)
Solid cancer	53 (16.1)	Basal + oral antidiabetic + correction	4 (1.2)
		Basal insulin only	3 (0.9)
		No treatment	4 (1.2)
Charlson comorbidity index, mean (SD)	5.82 (2.58)	Number of capillary glucose tests	8519
		Number of capillary glucose measurements per patient, median, IQR (25–75)	27 (16–49)
		Daily capillary glucose monitoring, median, IQR (25–75)	2.9 (2.0–4.0)
Ambulatory management, *n* (%)		Intrahospital management *n* (%)	
Glargine	96 (29.1)	Glargine	126 (38)
Degludec	24 (7.3)	Degludec	22 (6.7)
Xultophy/Soliqua	10 (3)	Xultophy/Soliqua	4 (1.2)
NPH	5 (1.5)	Detemir	1 (0.3)
Detemir	0 (0)	NPH	1 (0.3)
Rapid-acting analogs	92 (28)	Rapid-acting analogs	229 (69.3)
Regular insulin	5 (1.5)	Regular	137 (41.6)
Metformin	167 (50.8)	DPP4i	24 (7.3)
DPP4i	93 (28.2)	SGLT2i	12 (3.6)
SGLT2i	51 (15.5)	Metformin	8 (2.4)
Liraglutide	8 (2.4)	Liraglutide	2 (0.6)
Semaglutide	5 (1.5)	Semaglutide	1 (0.3)
Dulaglutide	2 (0.6)	Dulaglutide	0 (0)
Sulfonylureas	2 (0.6)	Sulfonylureas	0 (0)
Statins	212 (64.1)		
Beta-blockers	103 (31)		
Steroids	19 (5.8)		
Hospitalizations in the last 12 months, *n* (%)	118 (35.8)	Hospital treatment with glucocorticoids *n* (%)	39 (11.9)
1 prior hospitalization	76 (23)		
2 prior hospitalizations	22 (6.7)		
3 or more prior hospitalizations	20 (6)		

Abbreviations: AHF, acute heart failure; BMI, body mass index; CHF, chronic heart failure; CKD, chronic kidney disease; COPD, chronic pulmonary obstructive disease; DM, diabetes mellitus; DPP4i, dipeptidyl peptidase-4 inhibitors; ECV, cerebrovascular event; IAM, acute myocardial infarction; ICU, intensive care unit; IQR, interquartile range; SD, standard deviation; SGLT2i, sodium-glucose cotransporter 2 inhibitors.

^a^Grouping of admission reasons: acute complications of DM (hypoglycemia, hyperglycemia, ketoacidosis, or hyperosmolar state) and infections (urinary tract infection, pneumonia, skin and soft tissue infection, COVID-19, and others).

**Table 2:** Metrics of in-hospital glycemic control and comparative analysis according to in-hospital mortality.

	**All**	**In-hospital mortality**		**p** ^d^
		**Yes (** **n** **, %)**	**No (** **n** **, %)**	
HbA1c (%)^a^	7.3 (6.45–9.09)	7.97 (7.06–9.31)	7.22 (6.45–9.07)	0.200
HbA1c > 7.0% (*n*, %)	192 (58.2)	23 (7.0)	169 (51.2)	0.016
Average glucose level (mg/dL)^a^	145 (125–180)	186 (143–220)	143 (125–176)	<.001
Average of glucose outside of targets (%)^b^	91 (27.6)	17 (5.2)	74 (22.4)	<.001
SD^a^	43.3 (29.2–67.2)	57.5 (40.4–83.4)	41.6 (28.6–65.0)	0.007
CV^a^	29.5 (22.3–37.7)	33.8 (26.1–41.0)	28.9 (22.2–37.5)	0.062
CV > 36% (*n*, %)	109 (33)	14 (4.2)	95 (28.8)	0.068
TIR (%)^a^	58.0 (40.0–77.8)	41.0 (30.0–51.0)	60.0 (41.7–80.0)	<.001
TIR < 70% (*n*, %)^c^	214 (64.8%)	26 (7.9)	188 (57.0)	0.003
Hypoglycemia < 70 mg/dL level 1 (*n*, %)	95 (28.8)	10 (3.0)	85 (25.8)	0.478
Hypoglycemia < 54 mg/dL level 2 (*n*, %)	29 (8.8)	3 (0.9)	26 (7.9)	0.756

Abbreviations: CV, coefficient of variation (ratio of the standard deviation to the mean glucose)^30^; HbA1c, hemoglobin A1c; SD, standard deviation.

^a^Median (range).

^b^Average of glucose outside of targets (<100 or >180 mg/dl).

^c^TIR, time in range (100 to 180 mg/dl).

^d^Chi-square test (*X*^2^) for categorical variables and the Mann–Whitney test for continuous variables.

**Table 3:** Hospital outcomes.

**Outcomes**	**n** ** %**
Hospital mortality	29 (8.8)
Transfer to ICU	37 (11.2)
Readmission within 30 days	73 (22.1)
Delirium	106 (32.1)
Acute kidney injury	73 (22.1)
Hospital-acquired infections	56 (17.0)
Acute myocardial infarction	18 (5.5)
surgical site infection	4 (1.2)
Other infections in surgical patients	5 (1.5)

**Table 4:** Metrics of poor metabolic control and outcomes. Simple and adjusted logistic regression.

	**Average of capillary glucose measurements out of target range (**<100–>180 **mg/dL)**	**HbA1c (> 7%)**	**T** **I** **R** < 70%** (range between 100 and 180 mg/dL)**	%**CV** > 36	**Hypoglycemia (< 70 mg/dL)**
Outcomes					
Mortality	rOR 4.35 (1.98–9.52) *p* < .001	rOR 2.99 (1.19–7.56) *p* 0.016	rOR 5.21 (1.54–17.6) *p* 0.003	rOR 2.02 (0.93–4.36) *p* 0.068	rOR 1.34 (0.59–2.99) *p* 0.478
	aOR 5.97 (2.47–14.42) *p* < 0.001	aOR 4.05 (1.48–11.10) *p* 0.006	aOR 7.57 (2.09–27.36) *p* 0.002	aOR 1.92 (0.77–4.80) *p* 0.159	aOR 1.08 (0.44–2.64) *p* 0.851
Transfer to ICU	rOR 0.96 (0.44–2.09) *p* 0.937	rOR 1.37 (0.67–2.80) *p* 0.382	rOR 2.54 (1.08–5.98) *p* 0.028	rOR 1.64 (0.81–3.28) *p* 0.161	rOR 2.66 (1.33–5.33) *p* 0.005
	aOR 0.97 (0.35–2.65) *p* 0.956	aOR 1.66 (0.68–4.05) *p* 0.264	aOR 3.87 (1.28–11.64) *p* 0.016	aOR 2.22 (0.84–5.85) *p* 0.104	aOR 2.10 (0.83–5.28) *p* 0.113
Readmission within 30 days	rOR 0.49 (0.25–0.95) *p* 0.034	rOR 1.63 (0.94–2.81) *p* 0.079	rOR 1.14 (0.65–1.97) *p* 0.645	rOR 1.25 (0.728–2.16) *p* 0.415	rOR 1.39 (0.79–2.43) *p* 0.243
	aOR 0.50 (0.24–1.02) *p* 0.055	aOR 1.91 (1.06–3.42) *p* 0.03	aOR 1.16 (0.64–2.12) *p* 0.612	aOR 1.35 (0.72–2.53) *p* 0.341	aOR 1.31 (0.71–2.41) *p* 0.386
Delirium	rOR 1.58 (0.95–2.62) *p* 0.074	rOR 1.21 (0.75–1.94) *p* 0.426	rOR 1.08 (0.66–1.76) *p* 0.756	rOR 1.74 (1.07–2.81) *p* 0.024	rOR 1.99 (1.21–3.26) *p* 0.006
	aOR 2.40 (1.29–4.43) *p* 0.005	aOR 1.57 (0.90–2.71) *p* 0.105	aOR 1.34 (0.75–2.36) *p* 0.313	aOR 1.77 (0.97–3.24) *p* 0.06	aOR 1.54 (0.86–2.76) *p* 0.142
Acute kidney injury	rOR 1.92 (1.10–3.32) *p* 0.02	rOR 0.96 (0.57–1.64) *p* 0.899	rOR 1.34 (0.76–2.34) *p* 0.309	rOR 1.57 (0.919–2.69) *p* 0.097	rOR 1.63 (0.94–2.83) *p* 0.080
	aOR 2.34 (1.19–4.62) *p* 0.013	aOR 0.94 (0.50–1.78) *p* 0.864	aOR 1.27 (0.65–2.51) *p* 0.475	aOR 0.91 (0.46–1.80) *p* 0.796	aOR 1.37 (0.71–2.67) *p* 0.342
Hospital-acquired infections	rOR 1.44 (0.77–2.67) *p* 0.243	rOR 1.50 (0.81–2.73) *p* 0.189	rOR 1.30 (0.69–2.41) *p* 0.410	rOR 1.99 (1.11–3.58) *p* 0.019	rOR 2.14 (1.18–3.88) *p* 0.011
	aOR 1.50 (0.64–3.54) *p* 0.346	aOR 1.41 (0.62–3.17) p 0.403	aOR 1.07 (0.44–2.61) p 0.872	aOR 1.32 (0.55–3.16) p 0.530	aOR 0.81 (0.34–1.94) p 0.644
Acute myocardial infarction	rOR 1.01 (0.35–2.92) *p* 0.984	rOR 1.14 (0.42–3.01) *p* 0.796	rOR 1.09 (0.39–2.98) *p* 0.868	rOR 1.01 (0.37–2.78) *p* 0.978	rOR 2.07 (0.79–5.41) *p* 0.131
	aOR 1.21 (0.38–3.82) *p* 0.741	aOR 1.48 (0.52–4.18) *p* 0.456	aOR 1.36 (0.46–4.04) *p* 0.576	aOR 1.01 (0.31–3.25) *p* 0.976	aOR 1.95 (0.67–5.69) *p* 0.218

*Note:* When evaluating for an average > 200 mg/dL, statistical significance was lost, rOR 0.59 (0.27–1.28) *p* 0.18.

Abbreviations: aOR, adjusted OR for age, BMI, type of diabetes, reason for admission, length of hospitalization, heart failure, coronary disease, chronic kidney disease, CHF, and COPD; rOR, raw odds ratio.

**Table 5:** Variables associated with prolonged hospital stay.

**Associated variables**	**Median**	**p** ** value**
Hypoglycemia Level 1	Median 8 vs. 12 days	*p* < 0.001
Intrahospital infection	Median 8 vs. 23.5 days	*p* < 0.001
Transfer to ICU	Median 9 vs. 18 days	*p* < 0.001
Renal injury	Median 8 vs. 11 days	*p* 0.003
Delirium	Median 8.5 vs. 12 days	*p* < 0.001

## Data Availability

All relevant data are contained within the paper and its supporting information files. The data underpinning this article cannot be publicly shared due to privacy concerns. However, the data may be made available following approval from an appropriate ethics committee and must be managed in accordance with applicable data protection and privacy regulations.

## Nomenclature

%CV: coefficient of glycemic variability BMI (body mass index)
CKD: chronic kidney disease
DM: diabetes mellitus
HbA1C: glycated hemoglobin
LOS: length of stay
SD: standard deviation
TIR: time in range

## Supporting Information

Supporting Information Additional supporting information can be found online in the Supporting Information section. Table S1 Frequency of Level 1 hypoglycemia (<70 mg/dL). Summary of Level 1 hypoglycemia episodes in patients, showing the number of cases and percentages. Table S2: Frequency of Level 2 hypoglycemia (<54 mg/dL). Displays the frequency of level 2 hypoglycemia in patients, indicating the number and percentage by category. Table S3: Variables associated with poor in-hospital metabolic control multivariate analysis of factors such as HbA1c, CKD, BMI, and cause of admission, including odds ratios and statistical significance.
