# Supplementary material for: Derived Time in Range and Other Metrics of Poor Glycemic Control Associated With Adverse Hospital Outcomes in Patients With Diabetes Mellitus Admitted to Non-ICU Wards at a Tertiary-Level Hospital in Colombia: A Cross-Sectional Study
Source: J Diabetes Res. 2024 Aug 27;2024:3451158. doi: 10.1155/2024/3451158 (PMC11371450; doi:10.1155/2024/3451158)
Supplement: Supporting Information — Additional supporting information can be found online in the Supporting Information section. Table S1 Frequency of Level 1 hypoglycemia (<70 mg/dL). Summary of Level 1 hypoglycemia episodes in patients, showing the number of cases and percentages. Table S2: Frequency of Level 2 hypoglycemia (<54 mg/dL). Displays the frequency of level 2 hypoglycemia in patients, indicating the number and percentage by category. Table S3: Variables associated with poor in-hospital metabolic control multivariate analysis of factors such as HbA1c, CKD, BMI, and cause of admission, including odds ratios and statistical significance. [file 3451158.f1.docx]

**[Supplementary Appendix](https://www.nejm.org/doi/suppl/10.1056/NEJMoa2028198/suppl_file/nejmoa2028198_appendix.pdf)**

Table S1. Frequency of level 1 hypoglycemia <70mg/dL

| **Hypoglycemia** | **Patients** | **Percentage** |
| --- | --- | --- |
| 0 | 235 | 71.2 % |
| 1 | 51 | 15.5 % |
| 2 | 17 | 5.2 % |
| 3 | 8 | 2.4 % |
| 4 or more | 19 | 5,7 % |

Table S2. Frequency of level 2 hypoglycemia <54mg/dL

| **Hypoglycemia** | **Patients** | **Percentage** |
| --- | --- | --- |
| 0 | 301 | 91.2 % |
| 1 | 15 | 4.5 % |
| 2 | 7 | 2.1 % |
| 3 or more | 7 | 2.1 % |

Table S3. Variables associated with poor in-hospital metabolic control. Multivariate analysis with OR (95% CI)

|  | Average of capillary glucose measurements outside the target range (100-180 mg/dL) | %CV >36 | TIR <70% | Hypoglycemia |
| --- | --- | --- | --- | --- |
| HbA1c | **OR 1.20 (1.07-1.34)**  **P 0.002** | **OR 1.31 (1.14-1.50)**  **P <0.001** | **OR 1.35 (1.14-1.60)**  **P <0.001** | **OR 1.19 (1.06-1.33)**  **P 0.002** |
| CKD | OR 1.22 (0.68-2.18)  P 0.49 | **OR 2.77 (1.53-5.02)**  **P <0.001** | **OR 2.12 (1.22-3.71)**  **P 0.008** | OR 1.29 (0.74-2.26)  P 0.36 |
| BMI | OR 1.04 (0.99-1.10)  P 0.056 | **OR 0.91 (0.86-0.97)**  **P 0.005** | OR 0.96 (0.91-1.01)  P 0.12 | OR 0.96 (0.91-1.02)  P 0.23 |
| Admission cause: Reference group 6 (1. Acute complications of DM. 2. Infectious. 3. COPD. 4. AMI, CHF, or CVD. 5. Surgeries. 6. Other). | **1-6 OR 4.28 (1.48-12.39) P 0.007**  2-6 OR 1.54 (0.74-3.19)  P 0.24  3-6 OR 0.54 (0.09-3.02)  P 0.48  4-6 OR 0.92 (0.36- 2.35)  P 0.86  5-6 OR 1.66 (0.60-4.56)  P 0.32 | **1-6 OR 4.78 (1.50-15.19)**  **P 0.008**  2-6 OR 1.91 (0.93-3.91 P 0.07  3-6 OR 0.28 (0.03-2.60)  P 0.26  4-6 OR 0.49 (0.18- 1.32)  P 0.16  5-6 OR 1.34. (0.44-3.99)  P 0.59 | **1-6 OR 9.63 (1.12-82.30)**  **P 0.038**  2-6 OR 1.90 (0.99-3.63 P 0.052  3-6 OR 3.21 (0.91-11.23)  P 0.06  4-6 OR 1.12 (0.52- 2.42)  P 0.76  5-6 OR 2.34. (0.89-6.14)  P 0.08 | 1-6 OR 1.21 (0.41-3.51)  P 0.72  2-6 OR 1.34 (0.67-2.68) P 0.40  3-6 OR 0.63 (0.11-3.48)  P 0.60  4-6 OR 0.87 (0.36- 2.10)  P 0.76  5-6 OR 1.82. (0.67-4.95)  P 0.23 |

CV = coefficient of glycemic variation, TIR = time in range. a) Adjusted for age, BMI, type of diabetes, coronary and cerebrovascular disease, CHF, COPD, CKD, cause for admission, and HbA1c. b) Group 1 presented the worst metrics with the highest median of in-hospital average (202 mg/dl) compared with the other groups. It was also the group with the highest percentage (68.7%) out of the target range on average, as well as the highest CV (40.5) and the lowest tim
